# *Moringa oleifera* Lam.ameliorates the muscles function recovery following an induced insult to the Sciatic nerve in a mouse model

**DOI:** 10.1002/fsn3.1620

**Published:** 2020-07-11

**Authors:** Aroona Razzaq, Shoaib Ahmad Malik, Farhan Saeed, Ali Imran, Azhar Rasul, Muhammad Qasim, Shamaila Zafar, Syed Kashif Shahid Kamran, Javeria Maqbool, Muhammad Imran, Ghulam Hussain, Muzzamal Hussain

**Affiliations:** ^1^ Neurochemicalbiology and Genetics Laboratory (NGL) Department of Physiology Faculty of Life Sciences Government College University Faisalabad Pakistan; ^2^ Department of Biochemistry Sargodha Medical College University of Sargodha Sargodha Pakistan; ^3^ Institute of Home and Food Sciences Faculty of Life Sciences Government College University Faisalabad Pakistan; ^4^ Department of Zoology Faculty of Life Sciences Government College University Faisalabad Pakistan; ^5^ Department of Bioinformatics and Biotechnology Faculty of Life Sciences Government College University Faisalabad Pakistan; ^6^ Faculty of Allied Health Sciences University Institute of Diet and Nutritional Sciences The University of Lahore Lahore Pakistan; ^7^ University of Gambia Serrekunda Gambia

**Keywords:** blood glucose, *Moringa oleifera*, motor and sensory functional recovery, peripheral nerve injury, phytochemicals

## Abstract

Peripheral nerve injury (PNI) is an incapacitating situation and has no effective therapy until now. We examined the possible role of crude leaves of *Moringa oleifera* Lam. at 200 mg/kg body weight in accelerating the functional regain in the sciatic nerve lesion induced mouse model (Adult male albino mice (BALB/c). Motor functions were evaluated by using the sciatic functional index, muscle mass, and muscle grip strength measurement, whereas the sensory functions were evaluated by using the hot plate test. Blood glucose levels and blood cell composition were also analyzed. We found that the *Moringa oleifera* crude leaves endorse the sensory and motor functions reclamation following the PNI with a statistically significant difference (*p* < .05). It also revitalizes the gastrocnemius muscle by mass restoration with glycemic management perspective. Conclusively, the crude powder of *Moringa oleifera* leaves exhibited a function restoration boosting property and further detailed studies for its application as a therapeutic agent are strongly recommended.

## Graphical abstract

Protective and therapeutic effect of moringa on nerve injury.

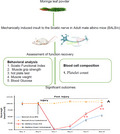

## 1 INTRODUCTION

Peripheral nerve injuries (PNIs) is very common and complex and impeding health factor in our society and remains incurable up to now. It may result from road traffic accidents, sharp lacerations, gunshot wounds, and various other physical traumas. Consequently, the affected nerves undergo an intricate pathological sequence and associated compromised sensory and motor functions. Though the peripheral nervous system is capable to exhibit a regenerative capability following the PNI, this phenomenon is quite slow and the impending muscular atrophy exacerbates the situation before the targeted organs’ re‐innervation (Aziz et al., 2019; Imran et al., 2019; Rasul et al., 2019) Despite incredible treatment approaches, complete functional retrieval still remains as a challenge to be resolved. The sluggish regeneration rates and the threatening muscular atrophy are the primary limitations in developing any novel therapeutic interventions against PNI. In order to cope with this need, scientists are exploring effective and affordable remedies to accelerate functional reclamation and endorse axonal regeneration (Hussain, Rasul, et al., 2018; Hussain, Zhang, et al., 2018). Meanwhile, plants and plant‐derived compounds are getting more consideration because of their health‐promoting properties (Hussain et al., 2019; Hussain, Rasul, et al., 2018; Hussain, Zhang, et al., 2018), as naturally occurring compounds present auspicious substitute therapeutic strategies for affected individuals.

Medicinal plants and herbs are a source of a plethora of phytochemicals that are demonstrated to be beneficial and effective in preventing, treating or improving different diseases and health conditions. *Moringa oleifera* Lam. (*M. oleifera*) is one of the richest sources of vitamins and minerals from the Moringaceae family. *Moringa has been widely utilized as folk medicine owing to its rich phytochemistry with special reference to* various phytoconstituents such as alkaloids, saponins, tannins, steroids, phenolic acids, glucosinolates, flavonoids, and terpenes. However, the presence of phenolic acids, glucosinolates, and flavonoids are responsible for its anticancer, neuroprotective, and antioxidant potential through various mechanistic routes. It is a native plant of some regions of the Himalayan mountains found in northwest Pakistan and India (Fahey, 2005). The extracts from *M. oleifera* evince various nutraceutical and pharmacological properties such as anti‐oxidant, anti‐cancerous, anti‐inflammatory, and neuroprotective and have been traditionally found to be a promising drug for various disorders such as cancer and liver diseases (Abdull Razis, Ibrahim, & Kntayya, 2014; Kooltheat et al., 2014). Interestingly, it mitigates memory impairment, age‐related dementia, neurodegeneration, Parkinson's disease, and Alzheimer's disease (Ganguly & Guha, 2008; Sutalangka, Wattanathorn, Muchimapura, & Thukham‐mee, 2013). Moreover, *M. oleifera* exhibits neurotrophic and neuroprotective properties as its leave extract stimulates and promotes neuronal outgrowth and survival both under normal toxic conditions (Hannan et al., 2014). However, the evidence concerning the effects of *M. oleifera* leaves in crude or extract forms on the nerve regeneration after an injury is scarce until now. Therefore, this study aimed to evaluate the potential role of crude leaf powder of *M. oleifera* in promoting the motor and sensory functions retrieval following a mechanically induced injury to the Sciatic nerve in a mouse model.

## 2 MATERIALS AND METHODS

### 2.1 Animals and study design for the experiment

Adult male albino mice (BALB/c) (*n* = 10) of the same weight (30 ± 4 g) and age (5–6 weeks) were provided by the department of physiology (Government College University Faisalabad). The mice were separated into two groups (Control group *n* = 5, Treatment group *n* = 5). All mice were given standard conditions of living with ad libitum food and water. They were housed in separate rodent plastic cages at ambient temperature and light/dark cycle as (24 ± 2°C; dark/light cycle). The study was approved by the Institutional Animal Care and Ethics Committee (ERC 254).

### 2.2 Plant collection and preparation

The leaves of *M. oleifera* were purchased from the local market of Faisalabad and identified by the botanist from the Department of Botany, Government College University Faisalabad. The leaves were shade dried and ground into powder as already described (Priyadarshani & Varma, 2014). Then, leaves powder was mixed in the normal chow diet of treatment group mice at the dose given in the literature as 200 mg/kg of body weight. The mice were given the privilege to consume an average diet intake of 5 g/animal; required to produce the biological effect according to the dose of *M. oleifera*. The average weight, drink, and food intake were measured throughout the period of the experiment (pre and post to the sciatic nerve injury) by adapting the standard procedures.

### 2.3 Sciatic nerve compression injury

The mice were subjected to the nerve crush after the acclimatization of one weak. For the purpose, they were anesthetized through intraperitoneal injection of ketamine (100 mg/kg BW) and xylazine (5 mg/kg BW) mixture (Ma et al., 2013). The incision site was shaved smoothly, and a fine cut over an extent of 2 cm along the proximal half of the fleck between the trochanter major and the knee joint was induced. The sciatic nerve was compressed (constant pressure) for 15 s by using a pair of forceps. It was made sure by visual observation that the nerve was perfectly compressed and the epineurium remained intact. Then, skin suturing was done (4‐0 stitches) and mice were permitted to recover (Halter et al., 2010; Hussain et al., 2013; Imran et al., 2019). After the induction of injury, mice were divided into two groups; the control group (*n* = 7) and the treatment group (*n* = 7).

### 2.4 Behavioral analysis

#### 2.4.1 Sciatic functional index

On all mice, walking track analysis was performed throughout the study as three times prior to surgery and then every third day after surgery. The plantar surface of mice was painted with the nontoxic ink and they were allowed to walk along the wooden track (50 cm × 7 cm). Approximately, five notable footprints were taken and the sciatic functional index (SFI) was measured with the following formula.

$$\text{SFI}=\left(-38.3\times -\frac{\text{EPL}-\text{NPL}}{\text{NPL}}\right)+\left(109.5\times \frac{\text{ETS}-\text{NTS}}{\text{NTS}}\right)\times \left(13.3\times \frac{\text{EIT}-\text{NIT}}{\text{NIT}}\right)-8.8$$

In the above formula, the distance between 1st and 5th toe is toe spread (TS), Print length (PL) is the distance from the top of the 3rd toe to heel, and the distance between 2nd and 4th toe is intermediary toe spread (IT) (Navarro, 2016).

#### 2.4.2 Muscle grip strength

Muscle grip strength measurement is an optimal parameter while assessing the motor functional recovery following the sciatic nerve lesion. It allows encountering the in vivo muscle strength force of mice and associated nerve regeneration capability as motor functions rehabilitation is indirectly associated with the rate of nerve regeneration. An average value of three readings was recorded by using muscle grip strength meter (Bioseb) for both hind limbs (Ipsilateral and contralateral). The results of the control and treatment group were compared with ensure the functional recovery.

#### 2.4.3 Thermal withdrawal threshold (hot plate test)

The regain of sensory functions was evaluated by using the thermal threshold with the hot plate apparatus (SCILOGEX MS7‐H550‐S LED digital 7 × 7 Hotplate stirrers). The hot plate test was performed by following the procedure as given previously (Aziz et al., 2019). In brief, the mouse was adapted to the nonfunctioning hot plate device for one minute before the actual experiment. Then, each mouse was adjusted in such a way that their operated hind paws were exposed to the hot surface of the functioning hot plate device (56 ± 2°C). The mouse was observed to elicit any response of jerk or lick in result to the thermal stimuli and this time was recorded as the hot plate latency. An average of three readings was taken as a final reading. Following such a response, the mouse was immediately removed from the device to avert any pathological intervention to the organ tissues.

#### 2.4.4 Muscle weight

The gastrocnemius muscle mass was measured in order to explore the extent of muscle atrophy as it is one of the most significant contributors in delaying the functional escalation. Following the muscle harvesting, the muscle mass of both groups (Ipsilateral legs) as normal chow group and *M. oleifera* chow group were measured and compared (Li et al., 2013; Tuffaha et al., 2016).

#### 2.4.5 Random blood glucose

Random blood glucose was measured in order to assess the contribution of glucose levels in exacerbating the pathological condition following the sciatic nerve injury. As a higher level of blood glucose initiates metabolism‐related pathological pathways at the site of PNI. A glucometer (Accu‐chek) was used to assess the blood glucose levels in both groups at different time intervals in the study according to the given procedure of (Asmat, Abad, & Ismail, 2016).

### 2.5 Blood cells composition analysis

The blood cell composition was measured by a hematological analyzer to evaluate the levels of various blood cell count either altered by the PNI or improved by the *M. oleifera* treatment.

## 3 RESULTS

### 3.1 Effect of *M. oleifera* on body weight and food intake

**FIGURE 1:**
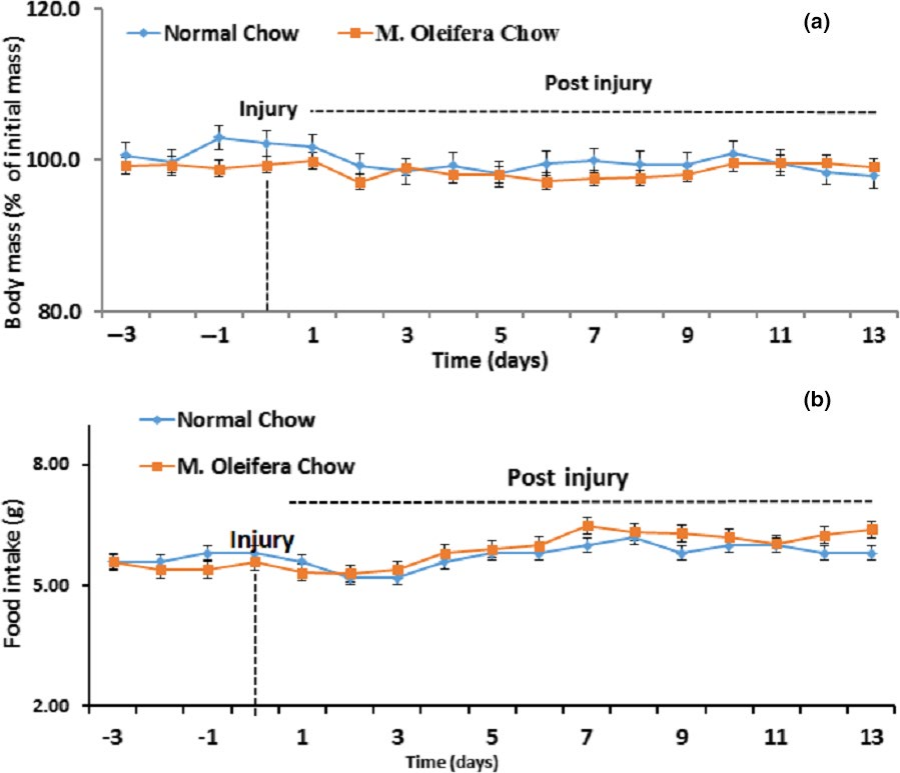
Effect of* Moringa oleifera *on body mass and food intake. (a): Time course of body mass in mice fed on normal chow (blue line, *n* = 5) and *M. oleifera* chow group (orange line, *n* = 5). Independent Student's *t*‐test showed the nonsignificant effect of time, nonsignificant effects of diet on the body mass. The statistical analysis is given as *p* = .421 at *p* = .005, *SD* = 3.74, *SEM* = 1.67 for normal chow group and *p* = *p *= .421 at *p* = .005, *SD* = 2.40, *SEM* = 1.07 for *M. oleifera* chow group with the confidence interval of 0.811–9.98. (b): Time course of body mass in mice fed on normal chow (blue line, *n* = 5) and *M. Oleifera* chow group (orange line, *n* = 5). Independent Student's *t*‐test showed the nonsignificant effect of time, nonsignificant effects of diet on food consumption. The statistical analysis at *p* = .005 is *p* = .300, *SD* = 2.74, *SEM* = 2.7 for normal chow group and *p* = .300, *SD* = 3.40, *SEM* = 5.0 for *Moringa oleifera* chow group with the confidence interval of 0.211–9.78

Body weight and food intake were measured in both groups, that is, normal chow group and *M. oleifera* chow group (Figure 1a,b) throughout the period of the experiment (pre and post to the sciatic nerve injury). We observed no statistical change (*p* = .461) in the diet intake and body mass in both groups even after the addition of *M. oleifera* in the mice diet and sciatic nerve lesion induction. Hence, it is speculated that *M. oleifera* did not modify the taste of diet and hence not hampered the diet intake.

### 3.2 Effect of *M. oleifera* on motor functions retrieval (SFI & Grip strength)

**FIGURE 2:**
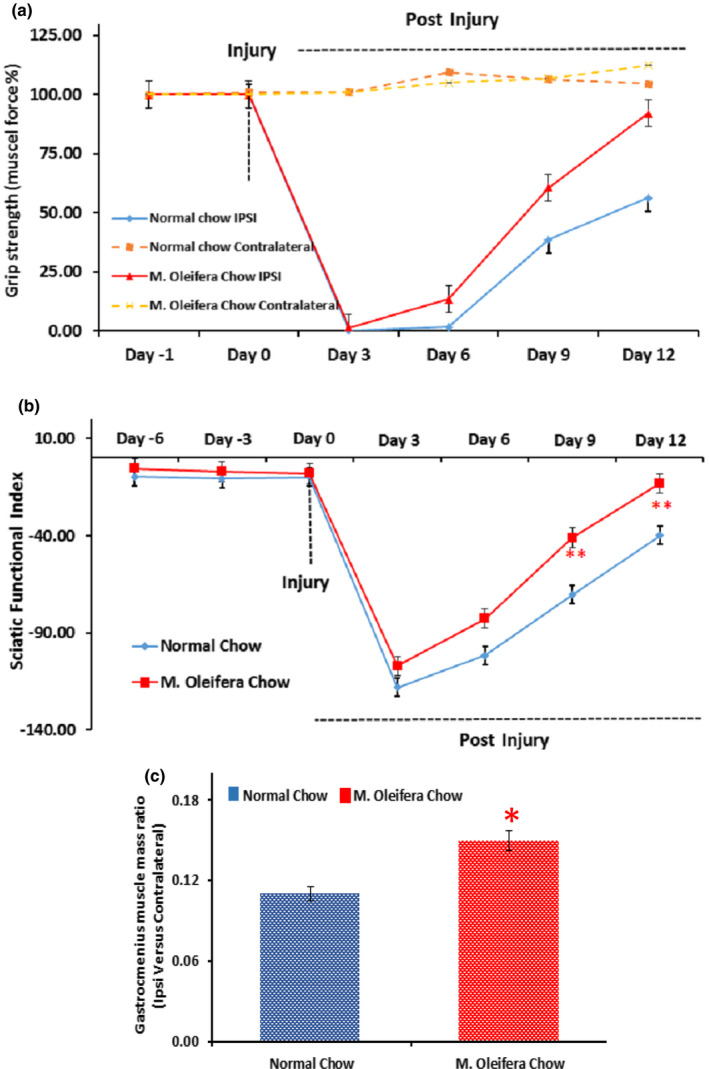
Effect of* Moringa oleifera *on motor functional recovery. (a): Time course of muscle grip strength (% of initial force) in mice fed on normal chow group (blue line, *n* = 5) and mice fed on *M. oleifera* chow group (red line, *n* = 5) after sciatic nerve crush. Measurements were acquired from both hind limbs as Ipsilateral (solid lines) and contralateral (dotted lines) to the mechanical insult of the sciatic nerve. Independent Student's *t*‐test showed a significant effect of time, significant effects of diet on muscle grip strength force. The statistical analysis is given as *p* = .002 at *p* = .005, *SD* = 9.34, *SEM* = 10.67 for normal chow group and *p* = *p* = **.002 at *p* = .005, *SD* = 4.40, *SEM* = 2.07 for *M. oleifera* chow group with the confidence interval of 18.811–10.98. (b) Time course of Sciatic functional index (SFI) in mice fed on normal chow group (blue line, *n* = 5) and mice fed on *M. oleifera* chow group (red line, *n* = 5). Measurements were acquired from both hind limbs as ipsilateral and contralateral to the mechanical insult of sciatic nerve. Independent Student's *t*‐test showed significant effect of time, significant effect of diet on SFI. The statistical analysis is given as *p *= **.029 at *p* = .005, *SD* = 10.54, *SEM* = 4.71 for normal chow group and *p *= **.029 at *p* = .005, *SD* = 11.29, *SEM* = 5.04 for *M. oleifera* chow group with the confidence interval of −34.08 to −2.37. (c): Time course of muscle mass restoration in mice fed on normal chow group (blue bar *n* = 5) and mice fed on *M. oleifera* chow group (red bar *n* = 5). Measurements were acquired from both hind limbs as Ipsilateral and contralateral (muscle mass ratio of both legs) to the mechanical insult of the sciatic nerve. Independent Student's *t*‐test showed a significant effect of time and a significant effect of diet on muscle mass restoration. The statistical analysis is given as *p *= *.04 at *p* = .005, *SD* = 1.54, *SEM* = 3.41 for normal chow group and *p *= *.04 at *p* = .005, *SD* = 1.29, *SEM* = 9.4 for *M. Oleifera* chow group with the confidence interval of 12.08 to −5.47

Following the sciatic nerve injury, there is a complete functional loss of motor neurons. Functional retrieval is directly connected to the rate of nerve regeneration, which is usually slow under ordinary situations. The SFI and Grip strength (% of initial force) were measured to evaluate the motor functional recovery. We found an early motor function recovery in the *M. oleifera* chow group (Figure 2a,b) with statistically significant differences (*p* < .05). Furthermore, the development of muscle atrophy is noteworthy, as shown by the decreased muscle mass, in normal chow group indicates the muscle denervation, whereas the muscle mass of Gastrocnemius muscle was recovered significantly in the *M. oleifera* chow group (Figure 2c).

### 3.3 Effect of *M. oleifera* on sensory functions retrieval

**FIGURE 3:**
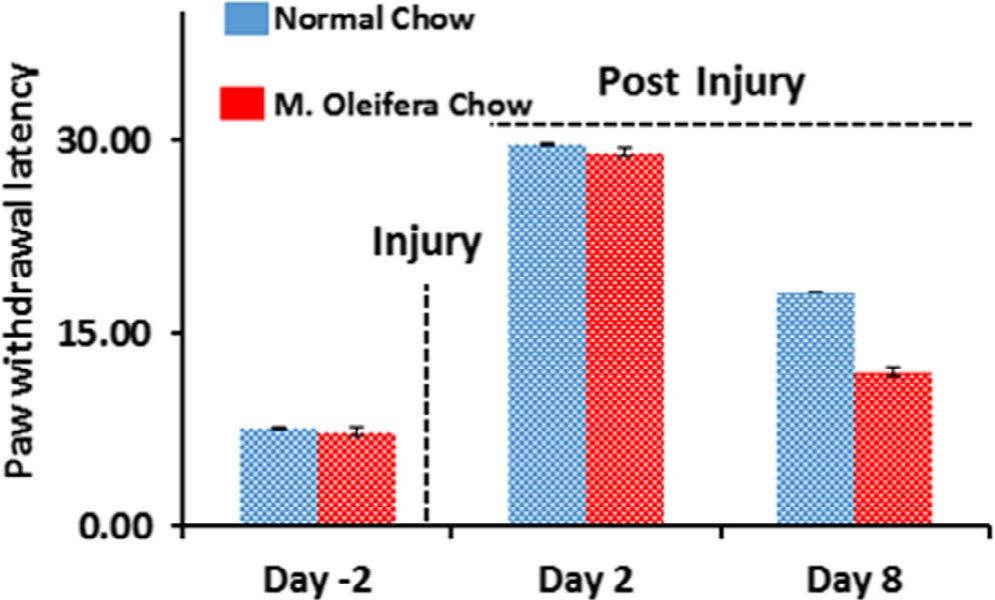
Effect of* Moringa oleifera *on motor functional recovery. (a) Time course of hot plate test (thermal nociception) in the mice fed on normal chow group (blue bar, *n* = 5) and the mice fed on *M. oleifera* chow group (red bar, *n* = 5) after sciatic nerve crush. Measurements were acquired from both groups’ operated hind limbs (Ipsilateral) before and after mechanical insult to the sciatic nerve. The hot plate test (Hot Plate Latency) results are expressed in seconds on different days (day −2, day 2 and 8). Independent Student's *t*‐test showed a significant effect of time, significant effects of diet on thermal nociception activity in the hot plate test. A significant difference was found between both groups as *p*= ***.000 at *p* = .005, *SD* = 0.78, *SEM* = 0.35 for *M. Oleifera* chow group with the confidence interval of 18.811–10.98

Following the sciatic nerve injury, both motor and sensory functions are compromised since the sciatic nerve is a mixed type of nerve. Therefore, measuring the sensory function is also equally important. The sensory function retrieval following the sciatic nerve lesion was evaluated by using the hot plate test (Figure 3). In the hot plate test, the paw withdrawal latency is indicative of nociception activity (Rasul et al., 2019). We found an early highly significant paw withdrawal response (*p* < .001), which shows sensory functions recovery, in the *M. oleifera* chow group. This ultimately indicates the effectiveness of *M. oleifera* in accelerating the rate of nociceptive activity retrieval after the sciatic nerve lesion.

### 3.4 Effect of *M. oleifera* on random blood glucose and blood cell measurement

**FIGURE 4:**
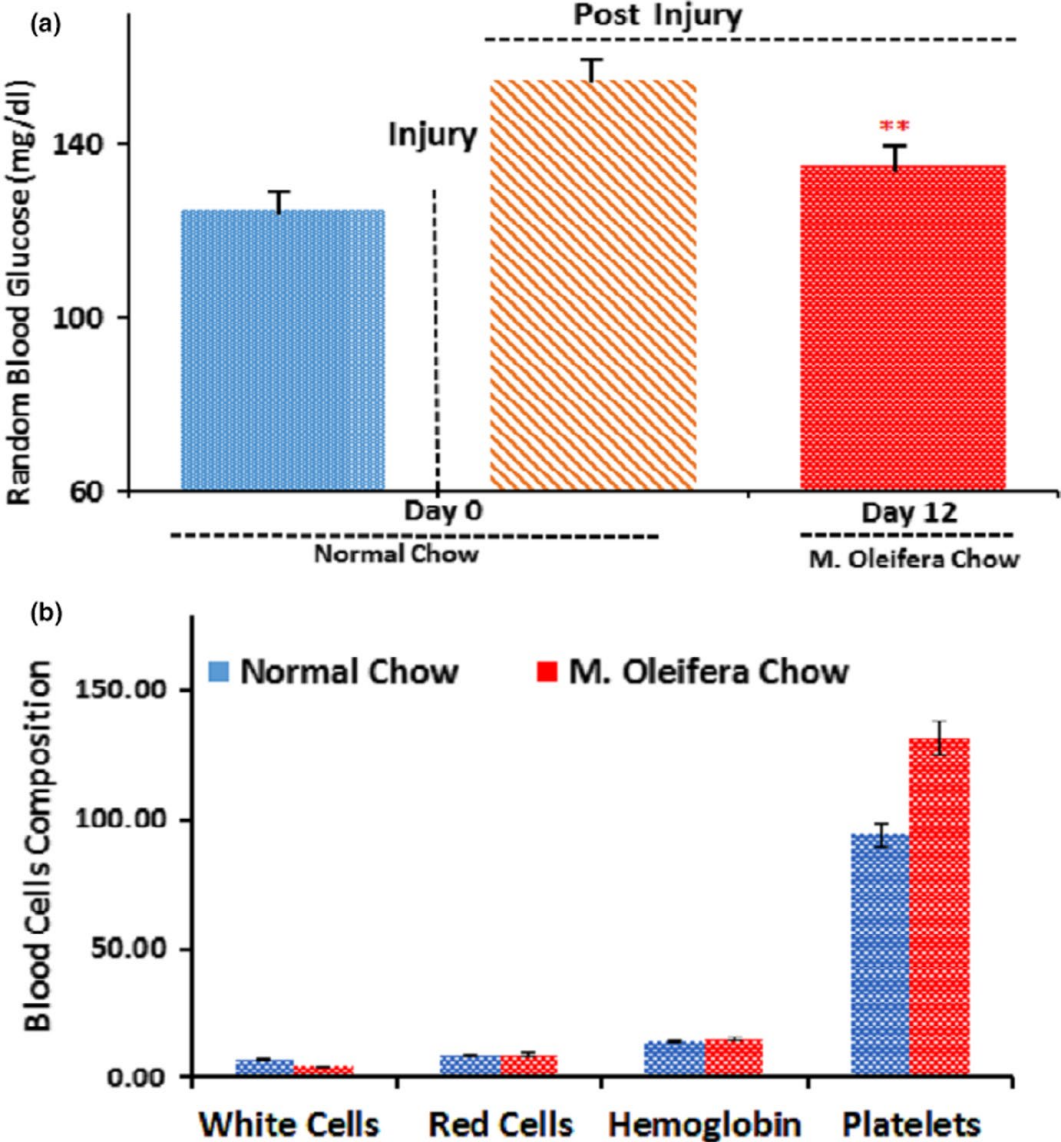
Effect of* Moringa oleifera* on blood glucose and blood cell composition. (a): Random blood glucose levels in mice fed on normal chow group (blue bar, *n* = 5) and mice fed on *M.* *oleifera* chow group (red bar, *n* = 5) after sciatic nerve crush. Independent Student's *t*‐test showed a significant effect of time, significant effects of diet on blood glucose levels. A significant difference was found between both groups as *p* = .003, *SD* = 4.53, *SEM* = 2.7 for *M. Oleifera* chow group with the confidence interval of 10.00–5.054. (b): Blood composition of both groups, normal chow group (*n* = 5) and *M. oleifera* chow group (*n* = 5). The 1st set of bars shows white blood cells level (103 μl) of both groups; statistically nonsignificant at (*p* = .161), 2nd set of bars show red blood cells level (106 μl) of both groups; statistically nonsignificant at (*p* = .386), 3rd set of bars show hemoglobin level (g/dl) of both groups; statistically nonsignificant at (*p* = .408), and 4th set of bars show platelets level (103 μl) of both groups; statistically nonsignificant at (*p* = .455)

The peripheral nerve function is affected by higher levels of blood glucose as deranged glucose metabolism initiates many pathological sequelae at the site of PNI. Therefore, measuring blood glucose levels is very effective while assessing the functional reclamation following the sciatic nerve injury (Nascimento, Pupe, & Cavalcanti, 2016). We found that random blood glucose levels were significantly reduced (*p* < .05) in the *M. oleifera* chow group (Figure 4a). *M. oleifera* has been previously found to enhance the platelet count, and here, we found that platelet level was also up‐regulated, though the results were not significant (Figure 4b). This might indicate the attenuation of an underlying pathological pathway of nerve injury, and thereby, it accelerates its regeneration.

## 4 DISCUSSION

This study was considered to inspect the possible role of *M. oleifera* as a novel agent in accelerating the nerve regeneration rate and associated functions regain in a mouse model of PNI. We observed that body mass and food intake were not altered in response to the addition of *M. oleifera* and induction of sciatic nerve lesion throughout the experiment. Moreover, we found that *M. oleifera* has the capability of promoting the motor and sensory functional recovery after the induction of sciatic nerve lesion to the mice. Based upon the severity and nature of the nerve injury, sensory and motor functions might compromise partially or completely. As the functional reclamation is directly associated with the nerve regeneration rate which is usually quite sluggish under conventional conditions. Here, we reported that *M. oleifera* causes an early reclamation of sensory and motor functions as shown in results with significant differences. The motor functions were found to be significant as measured by SFI (*p* = **.029) and grip strength (*p* = **.002). Whereas, the sensory functions, as measured by hot plate test (*p *= ***.000), were found to be retrieved earlier in the *M. oleifera* chow group. Furthermore, poor communication of the muscle due to the absence of basal stimuli causes the target muscle to undergo muscle atrophy. This phenomenon possesses additional burden at the site of nerve injury and thereby further decreasing the chances of functional retrieval (dos Santos et al., 2016). Meanwhile, the muscle atrophy also causes an additional delay in the regeneration of injured neurons as it halts the production of trophic factors and associated poor functional recovery (Li et al., 2013; Navarro, 2016; Tuffaha et al., 2016). Here, we found that the muscle atrophy in gastrocnemius muscle mass was reserved with a significant difference in the *M. oleifera* chow group (*p *= . *04) following the sciatic nerve injury. Taken altogether, these findings suggest that *M. oleifera* can accelerate the rate of function regain following an injury to the peripheral nervous system as well and these findings concomitantly endorse the already reported data regarding its benefits for the central nervous system (Singh & Navneet, 2018). It is already well documented by recent investigations that the neuronal dysfunctions and neurodegeneration could be improved by flavonoids intervention (Hussain, Rasul, et al., 2018; Hussain, Zhang, et al., 2018), we assume that the function promoting effects of *M. oleifera* might be attributed to the flavonoid class, but that requires further investigation.

Furthermore, in our study, we established that *M. oleifera* exhibits glucose‐lowering effects, which might be attributed to the anti‐diabetic effects of *M. oleifera* already reported (Patel, Ayaz, & Parikh, 2015). Thus, we hypothesize here that different phytochemicals in the *M. oleifera* modify the glucose metabolism machinery at cellular levels which eventually recovers the rate of nerve regeneration. Meanwhile, the higher levels of blood glucose cause additional pathological features at the PNI site by disturbing the physiological glucose metabolism and also imitates several pathological features [29]. Similarly, the hyperglycemic induces oxidative stress which is a well‐known factor as a dynamic power for the initiation of frequent clinical problems. This balance of anti‐oxidative and oxidative stress is very much sensitive to the glucose level since the minor elevated glucose level affects the entire oxidative system of the biological system. Here in PNI, this oxidative stress might serve as a key regulator in delaying functional retrieval (Asmat et al., 2016; Latini, Pereira, Couture, Campos, & Talbot, 2019; Menon et al., 2004), which could be overcome by the addition of *M. oleifera* extract in the diet. In this scenario, *M. oleifera* leaves contain flavonoids, such as quercetin and kaempferol, as the most potent antioxidants as well (Hussain, Rasul, et al., 2018; Hussain, Zhang, et al., 2018).

## 5 CONCLUSION

To sum up, this study provides evidence of the effectiveness of the Moringa leaves in the crude form against traumatic nerve injury. Based on the present findings and previous data, it can be suggested that this plant can prove an interesting and valuable target for the discovery of effective and affordable intervention against peripheral nerve injuries. Moreover, this study shows that oral administration of crude of *M. oleifera* does not affect the eating behavior as the body weight and eating pattern remained unmodified. Although these preliminary results are impregnated with positivity, however the further investigations to identify the actual bioactive constituent and its characterization is strongly recommended alongside the long‐term human‐based clinical trials ought to be conducted to apprehend the impact of these bioactive moieties and their possible mechanistic concerns in actual setting and their commercial adaptations.

## CONFLICT OF INTEREST

No conflict of interest stated from authors.

## ETHICAL APPROVAL

The study design and use of the animal model (Mouse) for the current project were approved by the Institutional Review Board (IRB) Government College University, Faisalabad, Pakistan.
